# Targeting Ca^2+^ and Mitochondrial Homeostasis by Antipsychotic Thioridazine in Leukemia Cells

**DOI:** 10.3390/life12101477

**Published:** 2022-09-23

**Authors:** Vivian W. R. Moraes, Vivian M. Santos, Eloah R. Suarez, Letícia S. Ferraz, Rayssa de Mello Lopes, Giuliana P. Mognol, Joana D. Campeiro, João A. Machado-Neto, Fabio D. Nascimento, Mirian A. F. Hayashi, Ivarne L. S. Tersariol, Donald D. Newmeyer, Tiago Rodrigues

**Affiliations:** 1 Center for Natural and Human Sciences, Federal University of ABC, Santo André 09210-580, SP, Brazil; 2 Department of Molecular Medicine, Scripps Research Institute, La Jolla, San Diego, CA 92037, USA; 3 Bluestar Genomics, San Diego, CA 92121, USA; 4 Department of Pharmacology, Federal University of São Paulo, São Paulo 04044-020, SP, Brazil; 5 Department of Pharmacology, Institute of Biomedical Sciences, University of São Paulo, São Paulo 05508-000, SP, Brazil; 6 Interdisciplinary Center of Biochemistry Investigation, University of Mogi das Cruzes, Mogi das Cruzes 08780-911, SP, Brazil; 7 Department of Molecular Biology, Federal University of São Paulo, São Paulo 04021-001, SP, Brazil; 8 La Jolla Institute for Allergy and Immunology, La Jolla, San Diego, CA 92037, USA

**Keywords:** apoptosis, calcium, endoplasmic reticulum stress, leukemia, mitochondria, phenothiazine

## Abstract

Mitochondria have pivotal roles in cellular physiology including energy metabolism, reactive oxygen species production, Ca^2+^ homeostasis, and apoptosis. Altered mitochondrial morphology and function is a common feature of cancer cells and the regulation of mitochondrial homeostasis has been identified as a key to the response to chemotherapeutic agents in human leukemias. Here, we explore the mechanistic aspects of cytotoxicity produced by thioridazine (TR), an antipsychotic drug that has been investigated for its anticancer potential in human leukemia cellular models. TR exerts selective cytotoxicity against human leukemia cells in vitro. A PCR array provided a general view of the expression of genes involved in cell death pathways. TR immediately produced a pulse of cytosolic Ca^2+^, followed by mitochondrial uptake, resulting in mitochondrial permeabilization, caspase 9/3 activation, endoplasmic reticulum stress, and apoptosis. Ca^2+^ chelators, thiol reducer dithiothreitol, or CHOP knockdown prevented TR-induced cell death. TR also exhibited potent cytotoxicity against BCL-2/BCL-xL-overexpressing leukemia cells. Additionally, previous studies have shown that TR exhibits potent antitumor activity in vivo in different solid tumor models. These findings show that TR induces a Ca^2+^-mediated apoptosis with involvement of mitochondrial permeabilization and ER stress in leukemia and it emphasizes the pharmacological potential of TR as an adjuvant in antitumor chemotherapy.

## 1. Introduction

Hematological neoplasms comprise a heterogeneous group of cancers that results from genetic abnormalities in the hematopoietic stem/progenitor compartment, including myeloproliferative neoplasms (MPN) and acute myeloid leukemia (AML) [1]. MPN may be stratified into Ph-positive or Ph-negative according to the presence of the BCR::ABL1 fusion gene, which is successfully targeted by tyrosine kinase inhibitors [2]. There is a trend of progression from MPN to AML, a situation that reflects a poor prognosis and a limitation of therapeutic options [3,4]. Evidence is accumulating that mitochondrial homeostasis plays a key role in the therapeutic response of these neoplasms [5,6]. In fact, mitochondria have been widely investigated as a target for the development/discovery of antitumor drugs, since they are intricately involved in energy metabolism, Ca^2+^ homeostasis, reactive oxygen species (ROS) production, and cell death [7,8,9]. In leukemia cells, the treatment with venetoclax (BCL2 inhibitor) impacts the tricarboxylic acid by inhibiting amino acid metabolism and contributing to the selective elimination of this cell population [10,11,12].

Phenothiazines (PTZs) are antipsychotic drugs used to treat schizophrenia [13]. Recently, an epidemiology-based retrospective study revealed that cancer incidence decreased in schizophrenia patients who had used PTZs, compared to other antipsychotic drugs [14]. In vitro studies showed that PTZs have antiproliferative effects in tumor cells [15], even in multidrug-resistant models [16]. Among PTZ derivatives, thioridazine (TR) ranks among the most cytotoxic, and the potency to induce cancer cell death correlates with the dissipation of mitochondrial membrane potential in hepatocarcinoma cells [17]. Additionally, TR sensitized glioma, renal, and breast cancer cells to apoptosis [18], and also targeted cancer stem cells by inhibiting dopamine receptors [19]. Another study screened a large chemical library of drugs approved by the FDA and PTZ featured among the most promising antitumor candidates by inducing apoptosis mediated by protein phosphatase 2A, but independent of their action on dopamine receptors [20]. Despite PTZ have been reported to induce apoptosis and exert a significant antitumor effect in different cancer models through several proposed pathways, the underlying molecular mechanisms involving calcium and mitochondrial alterations in that process remain not completely elucidated.

Here, we investigated the mechanisms of TR’s potent cytotoxicity, which was selective for tumor cells. The modulatory effect of TR on the expression of genes related to cell death pathways was screened by PCR arrays. We showed that TR-induced apoptosis in leukemia cells is mediated by Ca^2+^ and mitochondrial permeabilization, and it is accompanied by endoplasmic reticulum (ER) stress. TR exerted cytotoxicity even in leukemia cells overexpressing BCL2/BCL-xL antiapoptotic proteins. Furthermore, we observed a substantial antitumor effect of TR in vivo. Our findings thus highlight the pharmacological potential of PTZs in antitumor chemotherapy and help to clarify molecular mechanisms underlying their antitumor action.

## 2. Materials and Methods

### 2.1. Cell Culture

K562, K562 (shCHOP), HL60, HL60/BCL-2, HL60/BCL-xL, peripheral blood mononuclear cells (PBMCs), and mouse embryonic fibroblasts (MEFs) DKO (*Bax^−/−^/Bak^−/−^*) cells were grown in RPMI-1640 medium (Sigma-Aldrich, St. Louis, MO, USA), pH 7.2, supplemented with 10% fetal bovine serum (FBS) (Thermo Fisher Scientific, Waltham, MA, USA), 100 U/mL penicillin, and 100 µg/mL streptomycin (Thermo Fisher Scientific). K562 cells were acquired from Rio de Janeiro Cell Bank (BCRJ, Rio de Janeiro, Brazil), HL60 cell lines were donated by Prof. Gustavo Amarante-Mendes (Instituto de Ciências Biomédicas, Universidade de São Paulo), and MEFs came from La Jolla Institute of Allergy and Immunology (LIAI, San Diego, CA, USA). Cells were tested mycoplasma-free by indirect staining with Hoechst 33,258 (Thermo Fisher Scientific) and they were used during 4–8 passages after thawing the frozen stock. Cells were maintained at 37 °C in an atmosphere of 5% CO_2_ (Panasonic MCO-19AIC, Tokyo, Japan). PBMCs were isolated by centrifugation in the Ficoll-Paque gradient (GE Healthcare, Chicago, IL, USA) and stimulated with 5.0 mg/mL phytohemagglutinin for the assay. All procedures were performed in the dark to exclude the well-characterized photochemical effects of PTZs [21].

### 2.2. Cell Death Pathway Finder RT^2^ Profiler PCR Array

K562 cells (1 × 10^5^ cells/mL) were treated with 15 μM TR for 6 h and centrifuged at 160× *g* for 10 min. Total RNA was extracted from the cell pellet using the RNeasy Mini Kit (Qiagen, Hilden, Germany), and the cDNA was obtained using the RT^2^ Easy First Strand Kit (Qiagen), following the manufacturer’s instructions for both kits. The expression of cell death-related genes after treatment with TR was performed using the RT^2^ Profiler PCR Array-Human Cell Death Pathway (Qiagen). The complete list of genes analyzed in this kit is presented in Table S1. cDNA samples were mixed with RT^2^ SYBR Green ROX qPCR Mastermix (Qiagen) and aliquoted to the wells of array plates. The real-time cycling program was performed in a Stratagene Mx3005P real-time PCR system (Santa Clara, CA, USA), with thermocycler parameters of 95 °C for 10 min, followed by 40 cycles of 95 °C for 15 s and 60 °C for 1 min. Expression profiles were obtained from three independent experiments. The threshold cycle (Ct) of each gene was determined and subsequently analyzed by RT^2^ Profiler PCR Array data analysis software (Qiagen). Target gene expression was normalized based on the expression of five housekeeping genes (ACTB, B2M, GAPDH, HPRT1, and RPLP0). Total cellular RNA was isolated using TRIzol reagent (Invitrogen, Thermo Fisher Scientific) according to the manufacturer’s instructions. Total RNA was subsequently treated with DNase I (Qiagen) and further purified using RNeasy Mini Kit (Qiagen). Next, 1.0 mg of high-quality total RNA (RNA integrity number = 0.7) was reverse-transcribed using the First Strand Synthesis Kit (Qiagen) and subsequently loaded onto the RT^2^ profiler array according to the manufacturer’s instructions. Qiagen’s online analysis tool was used to produce comparative heat maps, and fold change was calculated by determining the ratio of mRNA levels in TR-treated cells in relation to control values (untreated K562 cells) using the D threshold cycle (Ct) method (ΔΔCt). All data were normalized to an average of five housekeeping genes: GUSB, HPRT, HSP90AB1, GAPDH, and ACTB. PCR conditions involved holding for 10 min at 95 °C, followed by 45 cycles of 15 s at 95 °C and 60 s at 60 °C. The heatmap was prepared using multiple experiment viewer (MeV) 4.9.0 software. Networks for modulated genes were constructed using the GeneMANIA database (https://genemania.org/, accessed on 29 June 2022) and the main biological interactions and processes were indicated. 

### 2.3. Cell Viability

Cells (1.0 × 10^5^ cells/mL) were plated into 96-well microplates in the presence of TR (Sigma-Aldrich) at different conditions and ultimately diluted to 0.2 mL of complete RPMI medium. Cytotoxicity was estimated by MTT (Sigma-Aldrich) reduction and trypan blue (Thermo Fisher Scientific) exclusion assays. The percentage of viable cells was calculated to control cells. The half maximal effective concentration (EC_50_) was calculated as described elsewhere [17]. 

### 2.4. Annexin V-FITC/PI 

After incubation, cells were centrifuged and the pellet was suspended in binding buffer (140 mM NaCl, 2.5 mM CaCl_2_, 10 mM HEPES, pH 7.4). After adding 3.0 µL Annexin V-FITC (BD Biosciences, San Jose, CA, USA) plus 5.0 µg/mL PI (BD Biosciences), samples were analyzed in a FACSCanto II cytometer (BD Biosciences) using FlowJo software (FlowJo, Ashland, OR, USA).

### 2.5. Caspase-9

Caspase-9 activity was measured using a spectrophotometric protease assay kit according to the manufacturer’s instructions (Thermo Fisher Scientific). Briefly, cell lysates were diluted with cell lysis buffer (2 mg/mL) and incubated with the reaction buffer and the caspase-9 substrate LEHD-*p*NA in a 96-well plate at 37 °C for 2 h in the dark. Afterward, the absorbance of each sample was measured at 405 nm by spectrophotometry.

### 2.6. Immunodetection of Active Caspase-3

K562 cells (1 × 10^5^ cells/mL) were incubated with 15 µM TR for 24 h, followed by fixation with 2% paraformaldehyde for 30 min and permeabilization with 0.01% saponin for 15 min at room temperature. Cells were collected and incubated with anti-active caspase-3 monoclonal antibody conjugated with FITC (BD Biosciences) for 40 min at 37 °C. Afterward, the cells were washed, and the fluorescence of 10,000 events were collected per sample in a FACS Canto II flow cytometer (BD Biosciences) and analyzed using FlowJo software (FlowJo).

### 2.7. Mitochondrial Membrane Potential

Mitochondrial transmembrane potential (ΔΨ) was kinetically monitored using 1.0 µM rhodamine 123 (Sigma-Aldrich) by fluorescent spectrophotometry, and 2.0 µM JC-1 or 5.0 nM TMRE (Thermo Fisher Scientific) by flow cytometry. For TMRE assay in MEF and HL60 cells, the fluorescence emission was monitored by flow cytometry using the PE-channel (488/575 nm, excitation/emission, respectively) in a FACS Canto II flow cytometer (BD Biosciences). For rhodamine 123 assay, ΔΨ was monitored at λ_ex_/λ_em_505/535 nm using a spectrofluorometer (F-2500, Hitachi, Tokyo, Japan). Potassium succinate (5.0 mM) was used as a substrate for the respiratory chain in the presence of 2.5 µM rotenone to inhibit complex I. The plasma membrane was permeabilized with 0.004% digitonin. The uncoupler CCCP was used as a control to obtain total ΔΨ dissipation. For the JC-1 assay, after 20 min of incubation with the dye, cells were washed twice with PBS, and the fluorescence emission was acquired at FITC and PE channels (FACS Canto II flow cytometer), with 50 µM CCCP used as a positive control. Data analysis was performed with FlowJo software (FlowJo).

### 2.8. Cytosolic and Mitochondrial Ca^2+^ Levels

For simultaneous imaging of mitochondrial and cytosolic Ca^2+^ signals, K562 cells (1 × 10^5^ cells/mL) incubated with 15 µM TR were loaded with 4.0 µM rhod-2/AM at 37 °C for 40 min, followed by incubation with 4.0 µM fluo-3/AM at 37 °C for another 40 min. Cells were loaded also with 5.0 nM TMRE (Thermo Fisher Scientific) to monitor ΔΨ concomitant to calcium levels. After being washed, double-loaded cells were simultaneously excited at 488 and 543 nm, and the fluorescence emission was monitored in live cells with a confocal laser scanning microscope (LSM-780 NLO, Carl Zeiss, Oberkochen, Germany), using a 600-nm long bandpass emission filter for rhod-2 and 500–550 nm for fluo-3. Images were processed using LSM 780 software (Carl Zeiss) and Image J (NIH, Bethesda, MD, USA).

### 2.9. Reactive Oxygen Species (ROS)

Intracellular ROS production was monitored using the cell-permeable chloromethyl derivative of 2′,7′-dichlorodihydrofluorescein (CM-H_2_DCFDA; Thermo Fisher Scientific). K562 cells (1.0 × 10^5^/mL) were loaded with 5.0 µM CM-H_2_DCFDA for 20 min in a 96-well plate. After washing with PBS, DCF fluorescence was analyzed for 120 min at λ_ex_/λ_em_ 485/530 nm after 15 µM TR was added or with 2.5 mM EGTA plus TR in a multi-well fluorescent microplate reader (Applied Biosystems, Bedford, MA, USA). As a positive control, 2.0 mM *t*-BOOH was used.

### 2.10. Glutathione and Reduced Protein Thiol Groups

Protein thiol groups (–SH) were quantified using 5,5′-dithiobis(2-nitrobenzoic) acid (DTNB, Ellman’s reagent). After 1.0 × 10^5^/mL K562 cells were incubated with 15 µM TR or 2.0 mM *t*-BOOH for 24 h and centrifuged for 10 min at 700× *g*, the cell pellet was treated with 0.2 mL of 6% trichloroacetic acid for protein precipitation, and centrifuged at 6000× *g* for 15 min. The resultant pellet was suspended with 1.0 mL 0.5 M PBS (pH 7.6) and after the addition of 0.1 mM DTNB, absorbance was acquired at 412 nm, and the amount of thiol groups in the control (i.e., without TR, considered to be 100%) was calculated from ε = 13,600 M^−1^.cm^−1^. The same procedure was applied to quantify reduced thiol groups of mitochondrial proteins isolated from those cells, with mitochondrial isolation performed as previously described [22]. Reduced glutathione (GSH) levels were fluorometrically estimated using *o*-phthalaldehyde [23]. An aliquot (0.1 mL) of the supernatant obtained after acid precipitation was added to 1.9 mL of a buffer containing 0.1 mg/mL NaH_2_PO_4_ and 5.0 mM EGTA pH 8.0, followed by the addition of 0.05 mg/mL *o*-phthalaldehyde. Fluorescence was analyzed at λ_ex_/λ_em_350/420 nm using a multi-well fluorescent microplate reader (Applied Biosystems).

### 2.11. MOMP Assay

The translocation of BAX to mitochondria and the release of Omi indicating the occurrence of MOMP were analyzed simultaneously by flow cytometry in HeLa cells transfected with BAX-Venus and Omi-mCherry, as described by Llambi et al. [24]. Cells with high fluorescence were selected by sorting, plated (1.0 × 10^5^/well), and treated with different concentrations of TR for another 24 h. Afterward, trypsin-treated cells were collected by centrifugation and washed with PBS. The cells were incubated with 0.02% digitonin for 10 min in an ice bath, washed twice with PBS, and analyzed in a BD LSRII flow cytometer (BD Biosciences) using the yellow laser and a 620/10-nm filter for mCherry, and the blue laser and a 525/50-nm filter for Venus. FlowJo software was used to analyze the results. The number of events analyzed per sample was 10,000.

### 2.12. Detection of Intracellular Phosphorylated Proteins

The phosphorylation of JNK, ERK1/2, and p38 was detected by flow cytometry using monoclonal antibodies against the phosphorylated form of each protein. K562 cells were incubated with 10 or 15 µM TR for 24 h and fixed with 2% paraformaldehyde for 10 min at 37 °C. Afterward, cells remained on the ice for 1 min and were permeabilized with 90% methanol on ice for 30 min, followed by washing with PBS containing 0.5% BSA. After blockading with PBS–BSA solution for 10 min at room temperature, cells were incubated with the specific antibody for the phosphorylated forms of JNK, ERK1/2, and p38−Alexa Fluor 488 anti-ERK1/2 pT202/pY204, Alexa Fluor 647 anti-JNK pT183/pY185, and Alexa Fluor 488 anti-p38 MAPK pT180/pY182, respectively (BD Biosciences), for 45 min in the dark at room temperature. After being washed, samples were centrifuged and suspended in PBS, 10,000 events were counted, and fluorescence was detected with a FACS Canto II flow cytometer (BD Biosciences). Analyses were performed using FlowJo software (FlowJo). As positive controls for protein activation, cells were exposed to 50 nM phorbol 12-myristate 13-acetate or 10 µg/mL anisomycin for 10 min.

### 2.13. Western Blotting

K562 or K562 shCHOP cells (1.0 × 10^5^ cells/mL) were plated in RPMI-1640 medium with FBS 10% in the presence or absence of 15 µM TR for 24 h. Samples for Western blot were prepared as previously described [25]. After protein transfer (Trans-Blot Turbo Transfer System, Bio-Rad Laboratories, Hercules, CA, USA), membranes were blocked using 2.5% BSA in Tris-buffered saline (TBS) for 1 h and incubated overnight at 4 °C with the following primary antibodies at 1:1000 dilution. Antibodies acquired from Cell Signaling Technology (Danvers, MA, USA) were anti-β-actin (#3700), anti-BIM (#2933), anti-BCL2 (#15071), anti-BCL-XL (#2764), ant-BAX (#5023), anti-CHOP (#2895), anti-IRE1α (#3294), anti-PERK (#5683), anti-CHOP (#2895), and IRE1α (#3294). Antibody anti-MCL1 (#559027) and anti-BAX (#556467) came from BD Biosciences (San Jose, CA, USA), and anti-phospho-PERK Thr 981 (#32577) from Santa Cruz Biotechnology (Dallas, TX, USA). After washing, membranes were incubated with anti-rabbit or anti-mouse HRP-conjugated secondary antibodies (Cell Signaling Technology) at 1:10,000 in blocking buffer for 1 h and washed 3 times with TBST buffer. After Western blot revelation with enhanced chemiluminescence SuperSignal™ West Femto (Thermo Fisher Scientific), images were obtained with ChemiDoc™ MP Imaging System (Bio-Rad), and the band densitometry was done using Scion Image Software version 4.03 (Scion Corporation, Frederick, MD, USA), considering three independent experiments.

### 2.14. shRNA-Mediated Knockdown of CHOP Gene

CHOP gene silencing was performed with three lentiviral vectors (i.e., TRCN0000007263, TRCN0000364328, and TRCN0000364393) containing the shRNA CHOP sequences (Table S2) for human genes (Gene ID: 1649) cloned into plasmid pLKO.1 acquired from library shRNA MISSION^®^ TRC-Hs 1.0/2.0 (Sigma-Aldrich). All plasmids contained bacterial (ampicillin) and human (puromycin) antibiotic resistance genes.

### 2.15. Statistical Analyses

Quantitative data are presented here as the mean ± SD of at least three independent experiments, performed in triplicate. The distribution of each dataset was defined as parametric or nonparametric by the Kolmogorov–Smirnov test, and for the comparison of two groups, the Mann–Whitney or *t* test was used for nonparametric and parametric variables, respectively. For the comparison of more than two groups, one-way ANOVA followed by Tukey’s post hoc test was used. Prism 6.0 software (GraphPad Software Inc., San Diego, CA, USA) was used to perform data analysis. Statistical significance was defined at *p* < 0.05.

## 3. Results

### 3.1. Thioridazine Modulates the Expression of Apoptosis-Related Genes and Induces Apoptotic Cell Death in Human Leukemia K562 Cells

**Figure 1:**
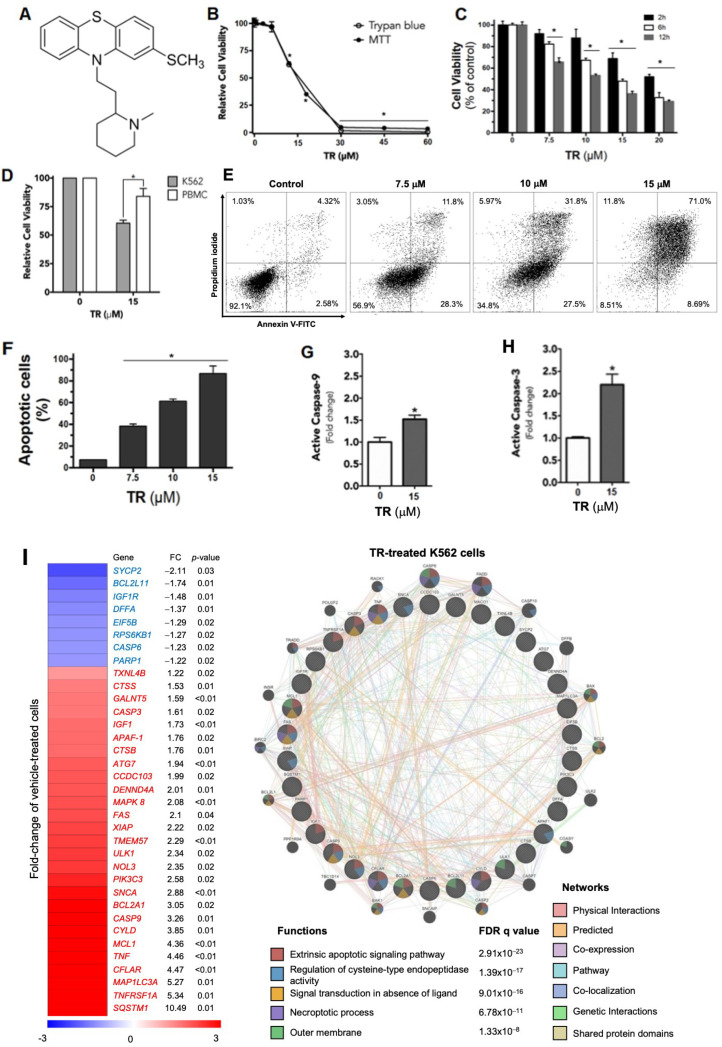

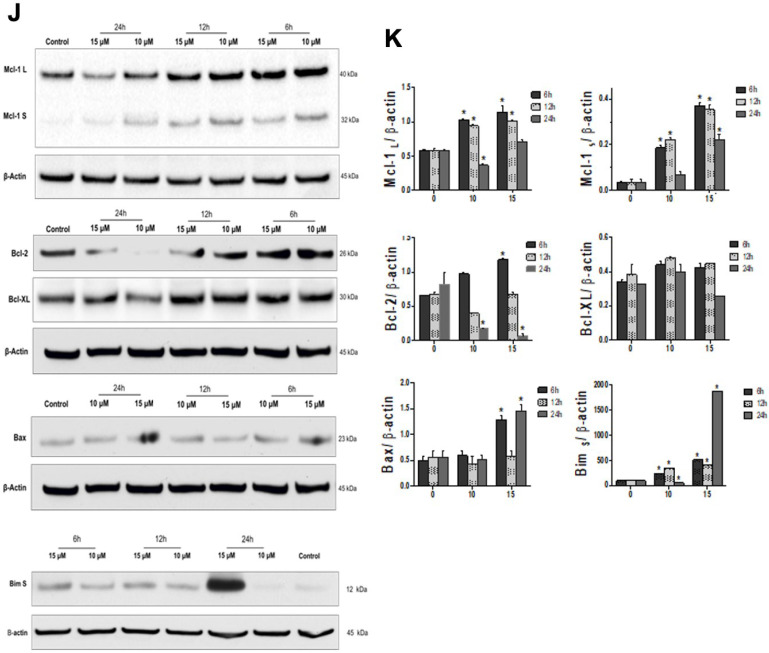
TR Induces Cell Death Selectively in Leukemia Cells. (**A**) Chemical structure of thioridazine (TR). (**B**) Viability of K562 cells in the presence of 0–60 µM TR incubated for 24 h using MTT reduction (empty circles) and trypan blue exclusion assays (full circles). (**C**) Viability of K562 cells in the presence of 0–20 µM TR incubated for 2 h (black bars), 6 h (white bars), and 12 h (grey bars) evaluated by MTT reduction assay. (**D**) Viability of K562 cells (grey bars) and PBMCs (white bars) treated with 15 µM TR for 24 h, assayed by MTT. In (**A**–**C**), data are expressed as percentages of viable cells (mean ± SD) calculated about control (i.e., without TR), considered to be 100%. (**E**) Representative flow cytometry dot plot of annexin V-FITC/PI doubled-stained in K562 cells incubated for 24 h with 15 µM TR. The number in each quadrant is the percentage of events. (**F**) Quantification of apoptotic annexin-V-positive cells (lower and upper right quadrants); data are expressed as percentages of apoptotic cells (mean ± SD). (**G**) The activity of caspase-9 in K562 cells incubated with 15 µM TR for 24 h determined spectrophotometrically. (**H**) Active caspase-3 in K562 cells upon incubation with 15 µM TR for 24 h by flow cytometry using a monoclonal antibody. Caspase-9 and -3 are expressed as fold changes (mean ± SD) about control. (**I**) Gene expression heatmap from qPCR array analysis of K562 treated with vehicle or 15 μM TR for 6 h. The mRNA levels were normalized to those of vehicle-treated K562 cells and calculated as fold changes in expression; genes with their expression significantly modulated in either direction are included in the heatmap. Network analysis for genes modulated by TR was constructed using the GeneMANIA database (https://genemania.org/, accessed on 29 June 2022). The upregulated and downregulated genes in the PCR array are illustrated as crosshatched circles, and the interacting genes included by the software modeling are indicated by circles without crosshatches. The main interactions between genes are indicated by colored lines and the five main cellular processes are described in the Figure. The *p* and FDR q values are indicated. (**J**) Modulation of the expression of Bcl-2 family proteins by TR (10 and 15 µM) incubated for 6, 12, and 24 h with K562 cells. Cells cultivated for 24 h in the absence of TR were used as control, and no significant differences were observed compared to 6 and 12 h (not shown). (**K**) Densitometry bands were quantified and normalized by their respective β-actin bands. Values are expressed as means ± SD. * Statistically different (*p* < 0.05).

We first measured the effects of TR (Figure 1A) on the viability of human leukemia cells using the MTT reduction test, which was confirmed by trypan blue exclusion assay (Figure 1B). TR produced a loss of viability of K562 cells in a concentration-dependent manner, with EC_50_ ~15 µM after 24 h of incubation (Figure 1B). As expected, the cytotoxic effect of TR depended upon the incubation time (Figure 1C), and it was selective for K562 cells compared with normal peripheral blood mononuclear cells (PBMC; Figure 1D). In the annexin V-FITC/propidium iodide (PI) (An/PI) assay, TR-treated cells displayed 61% An^+^/PI^−^ and 22% An^+^/PI^+^ staining, showing that TR-induced cell death occurred through apoptosis (Figure 1E,F). Consistent with apoptotic cell death, TR induced the activation of caspase-9 (Figure 1G) and caspase-3 (Figure 1H). Using a PCR array for cell death-related genes as an exploratory tool, a total of 35 of the 84 genes investigated were modulated after 6-h treatment by TR in K562 cells, including apoptosis- (*APAF-1, BCL2A1, BCL2L11, CASP3, CASP6, CASP9, CFLAR, DFFA, FAS, IGF1R, MCL1, NOL3, TNFRSF1A,* and *XIAP*), autophagy- (*ATG7, CTSB, CTSS, IGF1, MAP1LC3A, MAPK8, PIK3C3, RPS6KB1, SNCA, SQSTM1, TNF,* and *ULK1*), and necrosis-related genes (*TXNL4B, CYLD, DENND4A, CCDC103, EIF5B, GALNT5, PARP1, SYCP2,* and *TMEM57*) (all *p* < 0.05). Network analysis using the GeneMANIA tool and the genes modulated in TR treatment revealed involvement of the processes related to the extrinsic apoptotic signaling pathway, regulation of cysteine-type endopeptidase activity, signal transduction in absence of ligand, necroptotic process, and outer membrane (all FDR q value < 0.05) (Figure 1I). TR increased the expression of proapoptotic genes, including peptidase-activating factor 1 (APAF-1), as well as caspase-3 (CASP3) and caspase-9 (CASP9). Those data, associated with increase in caspase-3 and -9 activities in the presence of TR (Figure 1G,H), show that triggering the intrinsic apoptotic pathway is an important mechanism for TR to induce cell death. We also observed the upregulation of the TNF, TNF receptor member 1 (TNF-R1), and TNF-R member 6 (FAS) genes, and enhanced expression of BAX, an important effector of the extrinsic apoptotic pathway in leukemia cells [26].

Because the results from PCR arrays showed that TR can alter the gene expression of Bcl-2 family members, we also investigated the protein expression of selected genes by Western blot (Figure 1I). After 6 h of incubation, TR induced an increase of the expression of antiapoptotic proteins BCL-2 and MCL-1, probably as a protective response against chemical injury. However, after 24 h of incubation, TR not only increased the protein levels of proapoptotic members BIM and BAX, but also decreased the level of antiapoptotic protein BCL-2.

### 3.2. TR Disrupts Cellular Ca^2+^ Homeostasis in K562 Cells, Leading to Apoptosis

**Figure 2:**
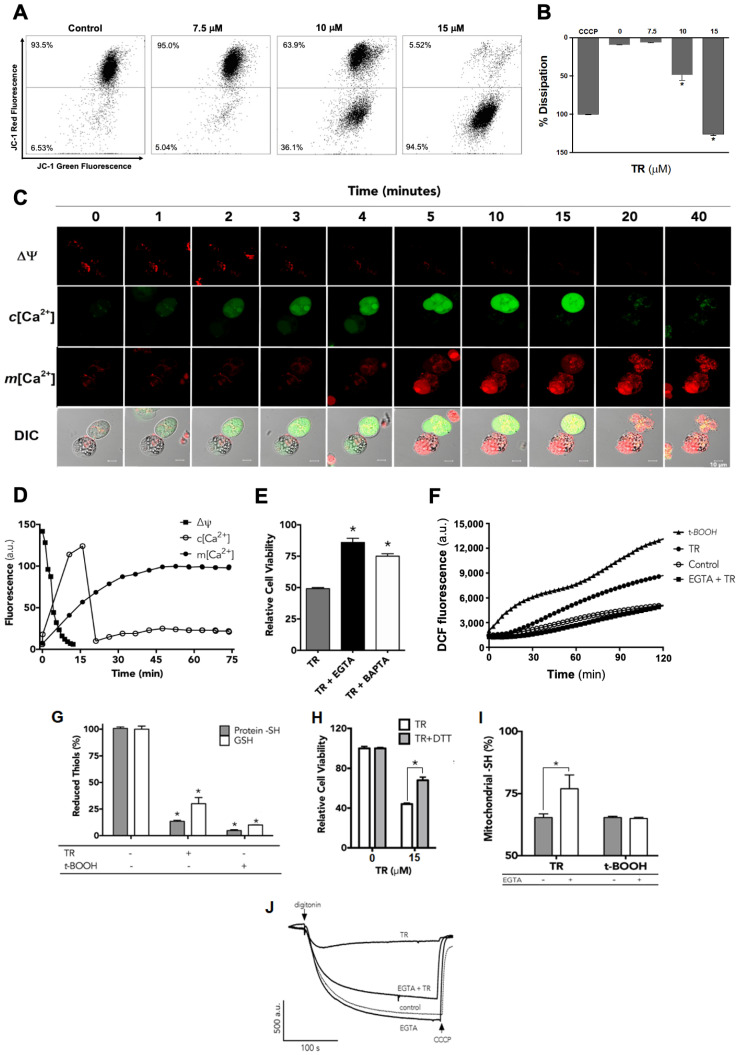
Increased Cytosolic Ca^2+^ Levels Induced by TR Result in Thiol Oxidation and Mitochondrial Permeabilization Associated with Cell Death. (**A**) Mitochondrial membrane potential (∆Ψ) of K562 cells incubated with TR (7.5, 10, and 15 µM) for 24 h was evaluated by flow cytometry using the JC-1 dye. Uncoupler CCCP was used as a positive control. (**B**) Quantification of the percentage of the dissipation of ΔΨ considering all replicates. Data are expressed as percentages of dissipation (mean ± SD). * Statistically different from the control (*p* < 0.05). (**C**) Representative images of the temporal evaluation of effects of 15 µM TR on ∆Ψ and both cytosolic and mitochondrial Ca^2+^ levels. K562 cells were loaded with rhod-2/AM (red) and fluo-3/AM (green) for simultaneous Ca^2+^ measurement by confocal microscopy. (**D**) Effect of 2.5 mM EGTA or 10 µM BAPTA-AM on the cytotoxicity of 15 µM TR incubated for 24 h with K562 cells. Data are expressed as percentages of control, considered to be 100% (mean ± SD). * Statistically different from TR (*p* < 0.05). (**E**) Representative traces of the effect of 2.5 mM EGTA on the TR-induced ΔΨ dissipation in digitonin-permeabilized K562 cells loaded with rhodamine 123. (**F**) Control (open circles), 15 µM TR (filled circles), 2.5 mM EGTA plus 15 µM TR (filled squares), and 2.0 mM *t*-BOOH (filled squares) were added to K562 cell suspension, and changes in the DCF fluorescence were recorded in real time. (**G**) Oxidation of reduced thiol groups of proteins (gray bars) and glutathione (white bars) by 15 µM TR in K562 cells after 24 h of incubation. *t*-BOOH was used as a positive control. * Statistically different from the control (*p* < 0.05). (**H**) Effect of 1.0 mM DTT on the cytotoxicity of 15 µM TR in K562 cells after 24 h of incubation. (**I**) Effect of 2.5 mM EGTA on the oxidation of reduced thiol groups of mitochondrial proteins induced by 15 µM TR or 2.0 mM *t*-BOOH after 24 h of incubation. (**J**) Impairment of the establishment of ∆Ψ by 15 µM TR in succinate-energized digitonin-permeabilized K562 cells and the protective effect of 2.5 mM EGTA.

Previous studies showed that PTZ derivatives triggered Ca^2+^ influx in *Saccharomyces cerevisiae* [27] and promoted Ca^2+^-dependent mitochondrial permeabilization associated with the release of cytochrome *c* in isolated rat liver mitochondria [28]. Mitochondrial depolarization can often be a diagnostic sign of mitochondrial-dependent cell death, which involves a loss of the barrier function of the mitochondrial outer membrane (MOM) and the release of proapoptotic proteins to the cytosol, triggering apoptosis [29]. To assess the involvement of mitochondria in the TR-induced killing of K562 cells, we measured mitochondrial depolarization using the cell-permeant JC-1 dye. As illustrated in Figure 2A and the respective quantification (Figure 2B), after 24 h incubation, TR induced a concentration-dependent dissipation of ΔΨ, with the maximal effect obtained at 15 µM.

Next, to monitor the effect of TR on cellular Ca^2+^ homeostasis in real time, cells were loaded simultaneously with fluo-3/AM and rhod-2/AM, respectively, to monitor cytosolic and mitochondrial Ca^2+^ levels by confocal laser scanning microscopy. As depicted in Figure 2C, TR promoted a biphasic kinetic response: first, an increase in cytosolic Ca^2+^([Ca^2+^]*c*), followed by a decrease 20 min later due to the loss of plasma membrane integrity induced by TR, as observed in the DIC images. Simultaneously, a progressive increase in the mitochondrial calcium levels ([Ca^2+^]*m*) was observed, which suggests mitochondrial uptake of the cytosolic Ca^2+^. To test whether this TR-induced disruption of Ca^2+^ homeostasis was required for cell death, we preincubated K562 cells with EGTA or BAPTA-AM to chelate extracellular and intracellular Ca^2+^, respectively, and following TR addition, measured cell viability with the MTT assay. Both chelators prevented cell death (Figure 2E). We conclude that a disturbance of Ca^2+^ is essential for TR-induced cell death. Preincubating K562 cells with EGTA inhibited the TR-induced dissipation of ΔΨ in digitonin-permeabilized cells (Figure 2E), corroborating our hypothesis that the disruption of Ca^2+^ homeostasis leads to mitochondrial permeabilization and it was required for TR-induced cell death.

### 3.3. Increased Ca^2+^ Levels Resulted in ROS Production and Thiol Oxidation in TR-Treated K562 Cells

Ca^2+^ can stimulate the generation of ROS via its mitochondria-mediated effects [30]. To investigate whether that occurs in TR-treated cells, we loaded the fluorescent ROS indicator CM-H_2_DCFDA into cells and then measured the kinetics of dichlorofluorescein (DCF) fluorescence emission. TR promoted a sustained increase in the production of ROS (Figure 2F) and the preincubation with EGTA abolished this increase, showing that Ca^2+^ was required for TR-induced ROS production. Since drug-induced thiol oxidation of mitochondrial proteins have been related to mitochondrial permeabilization and cell death [28,31,32], the effects of TR on the redox state of thiol groups was investigated. In whole cells, TR elicited glutathione (GSH) and protein thiol oxidation (Figure 2G) and 1,4-dithiothreitol (DTT) presented a partial protective effect on cellular viability (Figure 2H). Additionally, the oxidation of mitochondrial thiol groups by TR was observed (Figure 2I), which was partially inhibited by EGTA when the oxidation was promoted by TR, but not by *t*-BOOH, suggesting that TR did not directly oxidize those groups and, instead, likely increased cytosolic Ca^2+^, which, in turn, augmented ROS that oxidized thiols. Also, TR was pre-incubated with K562 cells and the formation of ΔΨ was triggered by the selective permeabilization of plasma membrane with digitonin (0.001%) in the presence of 5 mM potassium succinate as respiratory substrate and the ΔΨ was kinetically recorded. TR abrogated ΔΨ formation and this effect was prevented by the Ca^2+^ chelating activity of EGTA, showing the role of Ca^2+^ in TR-induced mitochondrial permeabilization in K562 leukemia cells (Figure 2J).

### 3.4. Assessment of the Role of Mitochondrial Permeabilization via Bcl-2 Proteins in TR-Induced Cell Death

**Figure 3:**
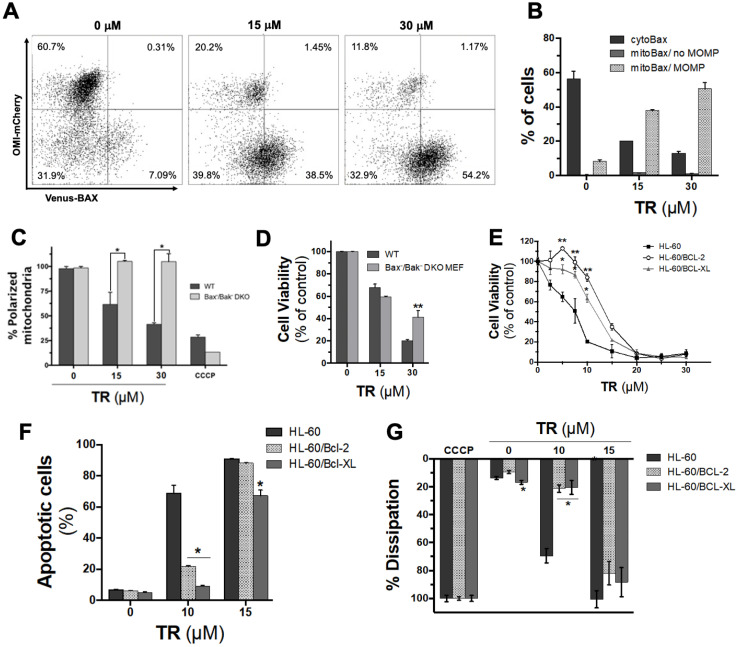
Mitochondrial Permeabilization in TR-Induced Cell Death and the Role of Bcl-2 Proteins. (**A**) Representative flow cytometry fluorescence dot plot of HeLa cells stably expressing Omi-mCherry and BAX-Venus incubated with 15 or 30 µM TR for 24 h. (**B**) Quantification of the decrease of Omi-mCherry and increase of BAX-Venus fluorescence indicating BAX translocation from the cytosol to the mitochondria resulting in MOMP, concomitant to the release of Omi/HtrA2 from the mitochondria to the cytosol during the apoptotic process. Effects of TR on ∆Ψ (**C**) and cell viability (**D**) of WT and *Bax*^−/−^*Bak*^−/−^ DKO MEFs after 24 h of incubation evaluated with TMRM and MTT, respectively. CCCP was used as a positive control. Data are expressed as percentages of viable cells (mean ± SD) calculated considering the control (i.e., without TR) as 100%. * Statistically different from WT cells (*p* < 0.05) (**E**) Effect of TR on the viability of HL60 human leukemia cells overexpressing BCL-2 or BCL-xL compared with WT HL60 after 24 h of incubation by MTT assay. Data are expressed as percentages of viable cells (mean ± SD) calculated considering the control as 100%. * Statistically different from WT cells (*p* < 0.05). (**F**) Quantification of annexin V-FITC-positive HL60 cells after 24 h of incubation with TR. Data are expressed as percentages of apoptotic cells (mean ± SD). * Statistically different from the control (*p* < 0.05). (**G**) Effects of TR on ∆Ψ of HL60 cells incubated with TR evaluated by flow cytometry with TMRM. CCCP was used as a positive control. * Statistically different from the control (*p* < 0.05).

It was reasonable then to hypothesize that Ca^2+^ release triggered ROS production and mitochondrial permeabilization. To test this, we used HeLa cells stably expressing Omi-mCherry and BAX-Venus [24]. When these cells are permeabilized using digitonin, Omi-mCherry and BAX-Venus molecules that had been present in the cytoplasm become released into the surrounding medium, while molecules located within mitochondria remain associated with cells. Therefore, flow cytometric measurement of mCherry-Omi and BAX-Venus fluorescence in these permeabilized cells allowed us to measure the extent of mitochondrial permeabilization in single cells, corresponding to a loss of mCherry-Omi fluorescence and an increase in BAX-Venus fluorescence. Figure 3A shows that TR induced a decrease in Omi-mCherry and an increase in BAX-Venus fluorescence. This result implies that BAX was translocated from the cytosol to the mitochondria, causing mitochondrial outer membrane permeabilization (MOMP), which resulted in the release of Omi/HtrA2 from the mitochondria to the cytosol, which in turn triggers apoptosis (Figure 3B). We conclude that TR induces MOMP, which then likely triggers caspase activation and apoptosis.

In apoptosis driven by the classical “intrinsic” pathway, MOMP depends on the proapoptotic effectors, BAX and BAK. To investigate whether this pathway is required for cell death caused by TR, we incubated wild-type (WT) and BAX/BAK double-knockout (*Bax*^−/−^*Bak*^−/−^ DKO) mouse embryonic fibroblasts (MEF) with TR and then measured both cell viability and changes in ΔΨ. The absence of BAX/BAK expression completely abolished the TR-induced dissipation of ΔΨ (Figure 3C). However, BAX/BAK deficiency had only a small effect at low TR concentration (15 µM) and a somewhat greater effect at a higher TR concentration (i.e., 30 µM, Figure 3D) in MEFs. This suggests that TR-induced cell death was mostly not mediated by the typical intrinsic mitochondrial pathway involving BAX and BAK, especially at low TR concentrations; at higher TR concentrations, intrinsic apoptosis may be activated to some extent. Because of our observation that TR induces a rapid cytoplasmic Ca^2+^ pulse and a sustained uptake of Ca^2+^ by mitochondria, and because Ca^2+^ chelators eliminated TR-induced cell death, it is reasonable to hypothesize that cell death is mostly caused by the Ca^2+^-induced mitochondrial permeability transition. Further investigation will be required to test this possibility.

Next, we tested the effects of antiapoptotic Bcl-2 family proteins BCL-2 and BCL-xL on TR-induced cell death, using human acute myeloid leukemia HL60 cells ectopically expressing BCL-2 or BCL-xL [33,34]. The overexpression of BCL-2 or BCL-xL somewhat decreased the cytotoxicity of TR (Figure 3E), as manifested in increased EC_50_ values (Table 1), as well as decreased percentages of apoptotic cells at 10 µM, but not 15 µM TR (Figure 3F). Additionally, the overexpression of BCL-2 or BCL-xL inhibited the dissipation of ΔΨ at low (10 µM) but not higher (15 µM) concentrations of TR (Figure 3G). Given the lack of protection afforded by BAX/BAK double knockout, we can speculate that the mild effects here of BCL-xL and especially BCL-2 could be to reduce Ca^2+^ release from the endoplasmic reticulum, rather than inhibit intrinsic apoptosis. In any case, it is important to note about the potential therapeutic uses of TR that moderate concentrations of TR were cytotoxic to tumor cells, despite the overexpression of these antiapoptotic BCL-2 family proteins. 

### 3.5. ER Stress Is Involved in TR-Induced Cell Death

**Figure 4:**
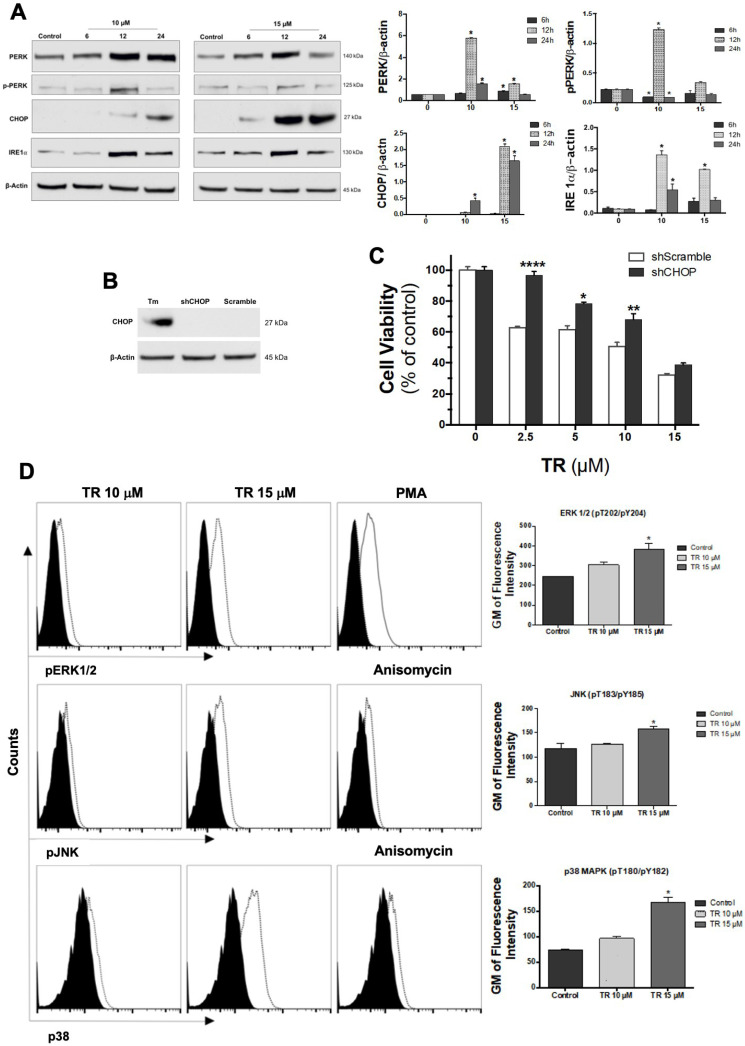
TR Induces ER Stress-Mediated Apoptosis. (**A**) Expression levels of UPR and ER stress proteins induced by 10 and 15 µM TR incubated with K562 cells for 12 and 24 h. Densitometry bands were quantified and normalized by their respective β-actin bands. Values are expressed as means ± SD. * Statistically different from the control (*p* < 0.05). (**B**) CHOP knockdown in K562 cells. CHOP expression was stimulated by 1.0 μg/mL tunicamycin (Tm) in the scramble and shCHOP cells. (**C**) Effects of increasing TR concentrations on shCHOPK562 cells after 24 h of incubation by MTT. Data are expressed as percentages of viable cells (mean ± SD) calculated considering the control (i.e., without TR) as 100%. * Statistically different from shScramble K562 cells (*p* < 0.05). (**D**) Expression levels of phosphorylated JNK and other stress-related signaling molecules ERK1/2 and p38 analysis by flow cytometry in K562 cells incubated with TR for 24 h. * Statistically different from the control (*p* < 0.05).

The transient Ca^2+^ disturbance we observe following TR treatment could reflect Ca^2+^ release from the ER. Possibly, this results from ER stress. Various cytotoxic stimuli, including drugs, can prompt the accumulation of unfolded proteins in the ER, thereby resulting in ER stress, which triggers an apoptotic cascade to eliminate the damaged cells [35,36]. We examined the involvement of ER stress in TR-induced cell death. As shown in Figure 4A, a major ER stress sensor protein, PERK, and its phosphorylated/active form, *p*-PERK, increased after treatment with TR during briefer incubations (i.e., 6 h and 12 h). One downstream target of PERK, namely, the transcriptional factor CHOP, the chief mediator of ER stress-induced cell death, was elevated after 24 h of incubation with TR. To test the requirement for this protein, we knocked down CHOP expression in K562 cells (Figure 4B) and then measured the effect of TR on cell viability (Figure 4C). As observed in Figure 4B, the expression of CHOP was undetectable in K562 cells (scramble). However, in the presence of the ER stressor tunicamycin (Tm), CHOP expression was significantly increased (left lane), which was then successfully knocked down in the shCHOP sample in the presence of Tm. Although CHOP knockdown (black bars) decreased the cytotoxicity of TR relative to the control (white bars), the effect was partial, suggesting that ER stress contributes to TR’s cytotoxicity only to a limited extent. Since the JNK signaling pathway is a downstream target of IRE1 and CHOP [37], we showed that TR promoted the phosphorylation of JNK and other stress-related signaling molecules (ERK1/2 and p38) (Figure 4D). Further research is required to evaluate the possibility that TR engages other mechanisms of ER stress that lead to Ca^2+^ release and cell death.

## 4. Discussion

Despite recent advances in the treatment of leukemia, drug resistance remains the major challenge to be overcome, which drives the constant search for new therapeutic options. Previous studies have shown that PTZs used in clinical practice presented antitumor activity in vitro against a variety of cancer cell lines, including neuroblastoma and glioma [38,39], lymphoma [40], leukemia [41], and prostate cancer cells [42]. In cancer stem cells, it was shown that TR antagonized dopaminergic D2 receptors [19], while in a study employing zebrafish as a model to screen drugs, Gutierrez et al. [20] proposed that perphenazine targets protein phosphatase 2A. Furthermore, a nanostructure-based system containing chlorpromazine also exerted cytotoxicity against a vincristine-resistant CML model [16], and a phase 1 clinical trial evaluated the combination of TR and cytarabine and revealed a reduction in blast levels [43]. Promethazine, a phenothiazine derivative with anti-histamine activity, also presented cytotoxicity in K562 cells [44]. Most published studies on cytotoxicity of phenothiazines in cancer models in vitro have reported the ability of these drugs to inhibit cell proliferation, migration, and invasiveness, to interfere with cell cycle, and to induce apoptosis in a variety of tumor cells [45]. It is clear that the cytotoxicity of TR obtained for tumor cells in vitro cannot be directly transposed to patients. In this regard, the EC_50_ calculated for TR in K562 cells is relatively higher than the maximal plasma concentration, according to the pharmacokinetic studies. Despite of this, the antitumor activity of TR has been shown in some in vivo models for solid tumors [46,47,48,49]. The administration of a drug for an animal or individual implicates in pharmacokinetic variables that impact the pharmacodynamics, and consequently the observed pharmacological effects. The maximal plasma concentration is only one of the parameters. In this regard, a recent clinical trial using thioridazine showed that the maximal plasma concentration in a cohort reached approximately 4.5 μM. However, at the 50 mg TR dose, the sum of circulating TR and its two active metabolites (2-sulfoxide and 2-sulfone) levels reached a 10 μM concentration, which is close to that observed in in vitro studies using leukemia cells [48]. Nevertheless, specific data about intracellular concentrations of TR in leukocytes are not found in the literature. A similar discussion has been observed about the cytotoxicity of phenothiazines against *Mycobacterium tuberculosis*. It was proposed that, despite the maximal plasma concentration reached by the phenothiazine intake being lower than that necessary to kill *M. tuberculosis* in vitro, drug bioaccumulation was showed in macrophages eliminating this infection in vivo [50,51]. Despite this, the molecular mechanisms underlying the cytotoxicity of clinically used PTZ derivatives in tumor cells remain to be clarified. The analysis of relevant literature has revealed that phenothiazines possess a multiplicity of targets, modulating several signaling pathways and cellular processes, although their effects on Ca^2+^ and mitochondrial homeostasis are not completely understood. Then, we examined the cytotoxicity of TR in vitro in a leukemia model, as well as the molecular pathways involved in TR-induced cell death.

We showed that TR produces an immediate increase of cytosolic Ca^2+^ levels, with a subsequent uptake by mitochondria, concomitant with ΔΨ dissipation. Together with the inhibitory effects of Ca^2+^ chelators (BAPTA-AM/EGTA) on TR-induced cell death, the results suggest that Ca^2+^ increase is an upstream event in TR-induced cell death that can trigger the downstream effects. A high-throughput drug screening identified TR as a highly selective drug for the MLL-AF6 rearrangement in AML, which is associated with a very poor prognosis. It was found that TR promoted the remodeling of the cytoskeleton in blasts, resulting in apoptosis-related Ca^2+^ influx [52]. Nevertheless, lingering questions include where and how TR mobilizes Ca^2+^ to the cytosol.

Aside from the involvement of mitochondria in TR-induced cell death, additionally, we showed that TR increased the expression levels of the primary ER stress sensors, PERK and IRE1α, and CHOP, the downstream target of PERK. CHOP induces the expression of GADD34 and increases levels of ERO1α, which results in the production of cytotoxic ROS [53]. As downstream signaling, the phosphorylation of JNK and other stress-related signaling molecules (ERK1/2 and p38) is in accordance with observed results from fluphenazine in MCF-7 breast cancer cells [54,55]. The knockdown of CHOP in K562 cells resulted in an attenuation of TR-promoted cytotoxicity, thereby demonstrating that ER stress contributes to TR-induced cell death. An activity-based protein profiling study also pointed to the contribution of ER stress and UPR for the cytotoxicity of chlorpromazine in glioblastoma cells [56].

As an intricate network, CHOP can modulate Bcl-2 proteins [57], inhibiting the transcription of the *BCL2* gene [58], and enhancing BIM expression [59]. Additionally, Bcl-2 proteins located in the ER regulate Ca^2+^ concentrations [60] and can regulate IRE1α [61]. Although BAX induced MOMP in TR-induced apoptosis, TR was able to induce death in *Bax*^−/−^*Bak*^−/−^ DKO cells, thereby indicating that other cellular processes/pathways contribute to cell death. The expression of Bcl-2 antiapoptotic proteins is upregulated in hematological malignancies, including leukemia [62], and several studies have been conducted to exploit those proteins as possible targets for drug development [63], with some inhibitors of the Bcl-2 family already being approved for leukemia treatment [64,65]. In that regard, TR was able to promote cell death even in myeloid leukemia cells overexpressing BCL-2 and BCL-xL, which is interesting considering that overexpression of antiapoptotic proteins is a resistance mechanism in leukemia treatment [66,67,68].

**Figure 5:**
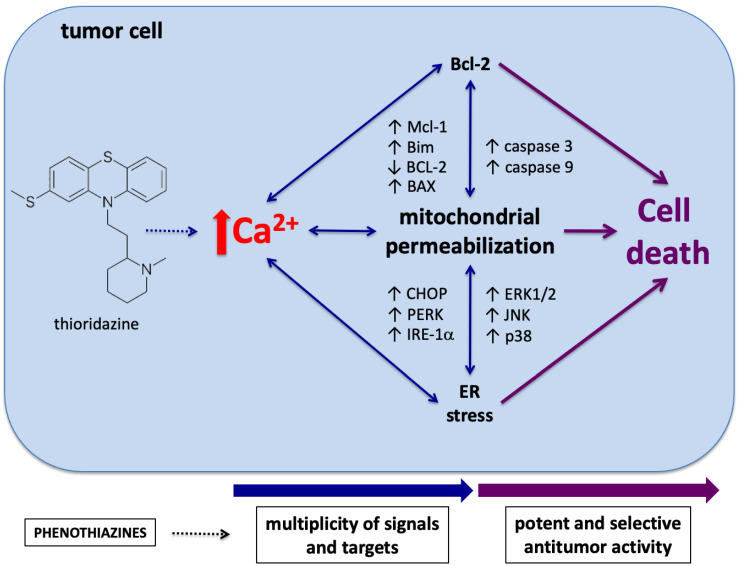
Schematic illustration showing multiple effects of TR in leukemia cells that culminate in cell death.

Taken together, our data demonstrate that TR induces complex, Ca^2+^-mediated cellular responses that result in mitochondrial permeabilization, ER stress, and apoptosis in K562 cells (Figure 5). Among other major results, TR presented selective efficacy against leukemia cells compared with peripheral blood mononuclear cells (PBMCs), and its cytotoxicity was observed even against leukemia model cells overexpressing antiapoptotic BCL-2/BCL-xL proteins. Beyond that, potent antitumor activity of TR has been shown in solid tumor models in vivo, such as melanoma [46,47] and glioblastoma [48,49]. Those results underscore that TR triggers a plethora of cellular effects involving different pathways and organelles that account for its cytotoxicity against tumor cells, pointing to TR as a promising candidate with pharmacological potential for antitumor chemotherapy.

## Figures and Tables

**Table 1:** Half maximal effective concentration (EC_50_) values for the cytotoxicity of thioridazine in leukemia HL60 cell lines.

Cell Line	EC_50_ (μM)
HL60	7.50 ± 0.02
HL60/BCL-2	13.40 ± 0.02
HL60/BCL-xL	11.60 ± 0.01

## Data Availability

Not applicable.

## Supplementary Materials

The following supporting information can be downloaded at: https://www.mdpi.com/article/10.3390/life12101477/s1, Table S1: List of 84 genes analyzed using the PCR Array RT2 Profiler–Human Cell Death Pathway Finder; Table S2: shRNA sequences used for CHOP knockdown in K562 cells.

## References

[1] Arber D.A., Orazi A., Hasserjian R., Thiele J., Borowitz M.J., Le Beau M.M., Bloomfield C.D., Cazzola M., Vardiman J.W. (2016). The 2016 Revision to the World Health Organization Classification of Myeloid Neoplasms and Acute Leukemia. Blood.

[2] Pizzi M., Croci G.A., Ruggeri M., Tabano S., Dei Tos A.P., Sabattini E., Gianelli U. (2021). The Classification of Myeloproliferative Neoplasms: Rationale, Historical Background and Future Perspectives with Focus on Unclassifiable Cases. Cancers.

[3] Dunbar A.J., Rampal R.K., Levine R. (2020). Leukemia Secondary to Myeloproliferative Neoplasms. Blood.

[4] Scherber R.M., Mesa R.A. (2020). Management of Challenging Myelofibrosis after JAK Inhibitor Failure and/or Progression. Blood Rev..

[5] Jayavelu A.K., Wolf S., Buettner F., Alexe G., Häupl B., Comoglio F., Schneider C., Doebele C., Fuhrmann D.C., Wagner S. (2022). The Proteogenomic Subtypes of Acute Myeloid Leukemia. Cancer Cell.

[6] Egan G., Khan D.H., Lee J.B., Mirali S., Zhang L., Schimmer A.D. (2021). Mitochondrial and Metabolic Pathways Regulate Nuclear Gene Expression to Control Differentiation, Stem Cell Function, and Immune Response in Leukemia. Cancer Discov..

[7] Olivas-Aguirre M., Pottosin I., Dobrovinskaya O. (2019). Mitochondria as Emerging Targets for Therapies against T Cell Acute Lymphoblastic Leukemia. J. Leukoc. Biol..

[8] Grasso D., Zampieri L.X., Capelôa T., Van De Velde J.A., Sonveaux P. (2020). Mitochondria in Cancer. Cell Stress.

[9] Rodrigues T., Ferraz L.S. (2020). Therapeutic Potential of Targeting Mitochondrial Dynamics in Cancer. Biochem. Pharmacol..

[10] Lagadinou E.D., Sach A., Callahan K., Rossi R.M., Neering S.J., Minhajuddin M., Ashton J.M., Pei S., Grose V., O’Dwyer K.M. (2013). BCL-2 Inhibition Targets Oxidative Phosphorylation and Selectively Eradicates Quiescent Human Leukemia Stem Cells. Cell Stem Cell.

[11] Pollyea D.A., Stevens B.M., Jones C.L., Winters A., Pei S., Minhajuddin M., D’Alessandro A., Culp-Hill R., Riemondy K.A., Gillen A.E. (2018). Venetoclax with Azacitidine Disrupts Energy Metabolism and Targets Leukemia Stem Cells in Patients with Acute Myeloid Leukemia. Nat. Med..

[12] Jones C.L., Stevens B.M., D’Alessandro A., Reisz J.A., Culp-Hill R., Nemkov T., Pei S., Khan N., Adane B., Ye H. (2018). Inhibition of Amino Acid Metabolism Selectively Targets Human Leukemia Stem Cells. Cancer Cell.

[13] Fenton M., Rathbone J., Reilly J., Sultana A. (2007). Thioridazine for Schizophrenia. Cochrane Database Syst. Rev..

[14] Mortensen P.B. (1989). The Incidence of Cancer in Schizophrenic Patients. J. Epidemiol. Community Health.

[15] Strobl J.S., Kirkwood K.L., Lantz T.K., Lewine M.A., Peterson V.A., Worley J.F. (1990). Inhibition of Human Breast Cancer Cell Proliferation in Tissue Culture by the Neuroleptic Agents Pimozide and Thioridazine. Cancer Res..

[16] De Mello J.C., Moraes V.W.R., Watashi C.M., Da Silva D.C., Cavalcanti L.P., Franco M.K.K.D., Yokaichiya F., De Araujo D.R., Rodrigues T. (2016). Enhancement of Chlorpromazine Antitumor Activity by Pluronics F127/L81 Nanostructured System against Human Multidrug Resistant Leukemia. Pharmacol. Res..

[17] de Faria P.A., Bettanin F., Cunha R.L.O.R., Paredes-Gamero E.J., Homem-de-Mello P., Nantes I.L., Rodrigues T. (2015). Cytotoxicity of Phenothiazine Derivatives Associated with Mitochondrial Dysfunction: A Structure-Activity Investigation. Toxicology.

[18] Min K.J., Seo B.R., Bae Y.C., Yoo Y.H., Kwon T.K. (2014). Antipsychotic Agent Thioridazine Sensitizes Renal Carcinoma Caki Cells to TRAIL-Induced Apoptosis through Reactive Oxygen Species-Mediated Inhibition of Akt Signaling and Downregulation of Mcl-1 and c-FLIP(L). Cell Death Dis..

[19] Sachlos E., Risueño R.M., Laronde S., Shapovalova Z., Lee J.H., Russell J., Malig M., McNicol J.D., Fiebig-Comyn A., Graham M. (2012). Identification of Drugs Including a Dopamine Receptor Antagonist That Selectively Target Cancer Stem Cells. Cell.

[20] Gutierrez A., Pan L., Groen R.W.J., Baleydier F., Kentsis A., Marineau J., Grebliunaite R., Kozakewich E., Reed C., Pflumio F. (2014). Phenothiazines Induce PP2A-Mediated Apoptosis in T Cell Acute Lymphoblastic Leukemia. J. Clin. Investig..

[21] Rodrigues T., Dos Santos C.G., Riposati A., Barbosa L.R.S., Di Mascio P., Itri R., Baptista M.S., Nascimento O.R., Nantes I.I. (2006). Photochemically Generated Stable Cation Radical of Phenothiazine Aggregates in Mildly Acid Buffered Solutions. J. Phys. Chem. B.

[22] Rodrigues T., Santos A.C., Pigoso A.A., Mingatto F.E., Uyemura S.A., Curti C. (2002). Thioridazine Interacts with the Membrane of Mitochondria Acquiring Antioxidant Activity toward Apoptosis—Potentially Implicated Mechanisms. Br. J. Pharmacol..

[23] Singh V., Gera R., Purohit M., Patnaik S., Ghosh D. (2017). Fluorometric Estimation of Glutathione in Cultured Microglial Cell Lysate. Bio. Protoc..

[24] Llambi F., Moldoveanu T., Tait S.W.G., Bouchier-Hayes L., Temirov J., McCormick L.L., Dillon C.P., Green D.R. (2011). A Unified Model of Mammalian BCL-2 Protein Family Interactions at the Mitochondria. Mol. Cell.

[25] Colturato-Kido C., Lopes R.M., Medeiros H.C.D., Costa C.A., Prado-Souza L.F.L., Ferraz L.S., Rodrigues T. (2021). Inhibition of Autophagy Enhances the Antitumor Effect of Thioridazine in Acute Lymphoblastic Leukemia Cells. Life.

[26] Han J., Goldstein L.A., Gastman B.R., Rabinovitz A., Wang G.Q., Fang B., Rabinowich H. (2004). Differential Involvement of Bax and Bak in TRAIL-Mediated Apoptosis of Leukemic T Cells. Leukemia.

[27] Eilam Y. (1983). Membrane Effects of Phenothiazines in Yeasts. I. Stimulation of Calcium and Potassium Fluxes. Biochim. Biophys. Acta.

[28] Cruz T.S., Faria P.A., Santana D.P., Ferreira J.C., Oliveira V., Nascimento O.R., Cerchiaro G., Curti C., Nantes I.L., Rodrigues T. (2010). On the Mechanisms of Phenothiazine-Induced Mitochondrial Permeability Transition: Thiol Oxidation, Strict Ca^2+^ Dependence, and Cyt c Release. Biochem. Pharmacol..

[29] Tait S.W.G., Green D.R. (2010). Mitochondria and Cell Death: Outer Membrane Permeabilization and Beyond. Nat. Rev. Mol. Cell Biol..

[30] Brookes P.S., Darley-Usmar V.M. (2004). Role of Calcium and Superoxide Dismutase in Sensitizing Mitochondria to Peroxynitrite-Induced Permeability Transition. Am. J. Physiol. Heart Circ. Physiol..

[31] Pessoto F.S., Faria P.A., Cunha R.L.O.R., Comasseto J.V., Rodrigues T., Nantes I.L. (2007). Organotellurane-Promoted Mitochondrial Permeability Transition Concomitant with Membrane Lipid Protection against Oxidation. Chem. Res. Toxicol..

[32] Moraes V.W.R., Caires A.C.F., Paredes-Gamero E.J., Rodrigues T. (2013). Organopalladium Compound 7b Targets Mitochondrial Thiols and Induces Caspase-Dependent Apoptosis in Human Myeloid Leukemia Cells. Cell Death Dis..

[33] Amarante-Mendes G.P., McGahon A.J., Nishioka W.K., Afar D.E.H., Witte O.N., Green D.R. (1998). Bcl-2-Independent Bcr-Abl-Mediated Resistance to Apoptosis: Protection Is Correlated with up Regulation of Bcl-XL. Oncogene.

[34] Brumatti G., Weinlich R., Chehab C.F., Yon M., Amarante-Mendes G.P. (2003). Comparison of the Anti-Apoptotic Effects of Bcr-Abl, Bcl-2 and Bcl-x(L) Following Diverse Apoptogenic Stimuli. FEBS Lett..

[35] Breckenridge D.G., Germain M., Mathai J.P., Nguyen M., Shore G.C. (2003). Regulation of Apoptosis by Endoplasmic Reticulum Pathways. Oncogene.

[36] Varadarajan S., Bampton E.T.W., Smalley J.L., Tanaka K., Caves R.E., Butterworth M., Wei J., Pellecchia M., Mitcheson J., Gant T.W. (2012). A Novel Cellular Stress Response Characterised by a Rapid Reorganisation of Membranes of the Endoplasmic Reticulum. Cell Death Differ..

[37] Tabas I., Ron D. (2011). Integrating the Mechanisms of Apoptosis Induced by Endoplasmic Reticulum Stress. Nat. Cell Biol..

[38] Gil-Ad I., Shtaif B., Levkovitz Y., Dayag M., Zeldich E., Weizman A. (2004). Characterization of Phenothiazine-Induced Apoptosis in Neuroblastoma and Glioma Cell Lines: Clinical Relevance and Possible Application for Brain-Derived Tumors. J. Mol. Neurosci..

[39] Pinheiro T., Otrocka M., Seashore-Ludlow B., Rraklli V., Holmberg J., Forsberg-Nilsson K., Simon A., Kirkham M. (2017). A Chemical Screen Identifies Trifluoperazine as an Inhibitor of Glioblastoma Growth. Biochem. Biophys. Res. Commun..

[40] Spengler G., Molnar J., Viveiros M., Amaral L. (2011). Thioridazine Induces Apoptosis of Multidrug-Resistant Mouse Lymphoma Cells Transfected with the Human ABCB1 and Inhibits the Expression of P-Glycoprotein. Anticancer Res..

[41] Zhelev Z., Ohba H., Bakalova R., Hadjimitova V., Ishikawa M., Shinohara Y., Baba Y. (2004). Phenothiazines Suppress Proliferation and Induce Apoptosis in Cultured Leukemic Cells without Any Influence on the Viability of Normal Lymphocytes. Phenothiazines and Leukemia. Cancer Chemother. Pharmacol..

[42] Csonka A., Spengler G., Martins A., Ocsovszki I., Christensen J.B., Hendricks O., Kristiansen J.E., Amaral L., Molnar J. (2013). Effect of Thioridazine Stereoisomers on the Drug Accumulation of Mouse Lymphoma and Human Prostate Cancer Cell Lines in Vitro. In Vivo.

[43] Aslostovar L., Boyd A.L., Almakadi M., Collins T.J., Leong D.P., Tirona R.G., Kim R.B., Julian J.A., Xenocostas A., Leber B. (2018). A Phase 1 Trial Evaluating Thioridazine in Combination with Cytarabine in Patients with Acute Myeloid Leukemia. Blood Adv..

[44] Medeiros H.C.D., Colturato-Kido C., Ferraz L.S., Costa C.A., Moraes V.W.R., Paredes-Gamero E.J., Tersariol I.L.S., Rodrigues T. (2020). AMPK Activation Induced by Promethazine Increases NOXA Expression and Beclin-1 Phosphorylation and Drives Autophagy-Associated Apoptosis in Chronic Myeloid Leukemia. Chem. Biol. Interact..

[45] Otręba M., Kośmider L. (2021). In Vitro Anticancer Activity of Fluphenazine, Perphenazine and Prochlorperazine. A Review. J. Appl. Toxicol..

[46] Jiang X., Chen Z., Shen G., Jiang Y., Wu L., Li X., Wang G., Yin T. (2018). Psychotropic Agent Thioridazine Elicits Potent in Vitro and in Vivo Anti-Melanoma Effects. Biomed. Pharmacother..

[47] Porta L.C., Campeiro J.D., Papa G.B., Oliveira E.B., Godinho R.O., Rodrigues T., Hayashi M.A.F. (2020). In Vivo Effects of the Association of the Psychoactive Phenotiazine Thioridazine on Antitumor Activity and Hind Limb Paralysis Induced by the Native Polypeptide Crotamine. Toxicon.

[48] Cheng H.W., Liang Y.H., Kuo Y.L., Chuu C.P., Lin C.Y., Lee M.H., Wu A.T.H., Yeh C.T., Chen E.T., Whang-Peng J. (2015). Identification of Thioridazine, an Antipsychotic Drug, as an Antiglioblastoma and Anticancer Stem Cell Agent Using Public Gene Expression Data. Cell Death Dis..

[49] Johannessen T.C., Hasan-Olive M.M., Zhu H., Denisova O., Grudic A., Latif M.A., Saed H., Varughese J.K., Røsland G.V., Yang N. (2019). Thioridazine Inhibits Autophagy and Sensitizes Glioblastoma Cells to Temozolomide. Int. J. Cancer.

[50] Crowle A.J., Douvas G.S., May M.H. (1992). Chlorpromazine: A Drug Potentially Useful for Treating Mycobacterial Infections. Chemotherapy.

[51] Ordway D., Viveiros M., Leandro C., Bettencourt R., Almeida J., Martins M., Kristiansen J.E., Molnar J., Amaral L. (2003). Clinical Concentrations of Thioridazine Kill Intracellular Multidrug-Resistant Mycobacterium Tuberculosis. Antimicrob. Agents Chemother..

[52] Tregnago C., Da Ros A., PorcùPorc E., Benetton M., Simonato M., Simula L., Borella G., Polato K., Minuzzo S., Borile G. (2020). Thioridazine Requires Calcium Influx to Induce MLL-AF6-Rearranged AML Cell Death. Blood Adv..

[53] Teske B.F., Fusakio M.E., Zhou D., Shan J., McClintick J.N., Kilberg M.S., Wek R.C. (2013). CHOP Induces Activating Transcription Factor 5 (ATF5) to Trigger Apoptosis in Response to Perturbations in Protein Homeostasis. Mol. Biol. Cell.

[54] Nordenberg J., Fenig E., Landau M., Weizman R., Weizman A. (1999). Effects of Psychotropic Drugs on Cell Proliferation and Differentiation. Biochem. Pharmacol..

[55] Otręba M., Sjölander J.J., Grøtli M., Sunnerhagen P. (2021). A Small Molecule Targeting Human MEK1/2 Enhances ERK and P38 Phosphorylation under Oxidative Stress or with Phenothiazines. Life.

[56] Matteoni S., Matarrese P., Ascione B., Ricci-Vitiani L., Pallini R., Villani V., Pace A., Paggi M.G., Abbruzzese C. (2021). Chlorpromazine Induces Cytotoxic Autophagy in Glioblastoma Cells via Endoplasmic Reticulum Stress and Unfolded Protein Response. J. Exp. Clin. Cancer Res..

[57] Rodriguez D., Rojas-Rivera D., Hetz C. (2011). Integrating Stress Signals at the Endoplasmic Reticulum: The BCL-2 Protein Family Rheostat. Biochim. Biophys. Acta.

[58] McCullough K.D., Martindale J.L., Klotz L.-O., Aw T.-Y., Holbrook N.J. (2001). Gadd153 Sensitizes Cells to Endoplasmic Reticulum Stress by Down-Regulating Bcl2 and Perturbing the Cellular Redox State. Mol. Cell Biol..

[59] Puthalakath H., O’Reilly L.A., Gunn P., Lee L., Kelly P.N., Huntington N.D., Hughes P.D., Michalak E.M., McKimm-Breschkin J., Motoyama N. (2007). ER Stress Triggers Apoptosis by Activating BH3-Only Protein Bim. Cell.

[60] Zong W.X., Li C., Hatzivassiliou G., Lindsten T., Yu Q.C., Yuan J., Thompson C.B. (2003). Bax and Bak Can Localize to the Endoplasmic Reticulum to Initiate Apoptosis. J. Cell Biol..

[61] Hetz C., Bernasconi P., Fisher J., Lee A.H., Bassik M.C., Antonsson B., Brandt G.S., Iwakoshi N.N., Schrinzel A., Glimcher L.H. (2006). Proapoptotic BAX and BAK Modulate the Unfolded Protein Response by a Direct Interaction with IRE1alpha. Science.

[62] Vogler M., Butterworth M., Majid A., Walewska R.J., Sun X.M., Dyer M.J.S., Cohen G.M. (2009). Concurrent Up-Regulation of BCL-XL and BCL2A1 Induces Approximately 1000-Fold Resistance to ABT-737 in Chronic Lymphocytic Leukemia. Blood.

[63] Perini G.F., Ribeiro G.N., Pinto Neto J.V., Campos L.T., Hamerschlak N. (2018). BCL-2 as Therapeutic Target for Hematological Malignancies. J. Hematol. Oncol..

[64] Ruefli-Brasse A., Reed J.C. (2017). Therapeutics Targeting Bcl-2 in Hematological Malignancies. Biochem. J..

[65] DiNardo C.D., Pratz K.W., Letai A., Jonas B.A., Wei A.H., Thirman M., Arellano M., Frattini M.G., Kantarjian H., Popovic R. (2018). Safety and Preliminary Efficacy of Venetoclax with Decitabine or Azacitidine in Elderly Patients with Previously Untreated Acute Myeloid Leukaemia: A Non-Randomised, Open-Label, Phase 1b Study. Lancet Oncol..

[66] Mehta S.V., Shukla S.N., Vora H.H. (2013). Overexpression of Bcl2 Protein Predicts Chemoresistance in Acute Myeloid Leukemia: Its Correlation with FLT3. Neoplasma.

[67] Zhou J.D., Zhang T.J., Xu Z.J., Gu Y., Ma J.C., Li X.X., Guo H., Wen X.M., Zhang W., Yang L. (2019). BCL2 Overexpression: Clinical Implication and Biological Insights in Acute Myeloid Leukemia. Diagn. Pathol..

[68] Ball S., Borthakur G. (2020). Apoptosis Targeted Therapies in Acute Myeloid Leukemia: An Update. Expert Rev. Hematol..

