# Supplementary material for: Targeting Ca2+ and Mitochondrial Homeostasis by Antipsychotic Thioridazine in Leukemia Cells
Source: Life (Basel). 2022 Sep 23;12(10):1477. doi: 10.3390/life12101477 (PMC9605445; doi:10.3390/life12101477)
Supplement: Supplementary file 1 [file life-12-01477-s001.zip › life-1899705-supplementary.pdf]

Table S1. List of 84 genes analyzed using the PCR Array RT² Profiler – Human Cell Death Pathway Finder.

**Pathway**	**Genes**
Apoptosis	ABL1, APAF1, ATP6V1G2, BAX, BCL2L11, BIRC2 (c-IAP2), CASP1 (ICE), CASP3, CASP6, CASP7, CASP9, CD40 (TNFRSF5), CD40LG (TNFSF5), CFLAR (CASPER), CYLD, DFFA, FAS (TNFRSF6), FASLG (TNFSF6), GADD45A, NOL3, SPATA2, SYCP2, TNF, TNFRSF1A, TNFRSF10A (TRAIL-R), TP53, AKT1, BCL2, BCL2A1 (Bfl-1/A1), BCL2L1 (BCL-X), BIRC3 (c-IAP1), CASP2, IGF1R, MCL1, TNFRSF11B, TRAF2, XIAP.
Autophagy	AKT1, APP, ATG12, ATG16L1, ATG3, ATG5, ATG7, BAX, BCL2, BCL2L1 (BCL-X), BECN1, CASP3, CTSB, CTSS, ESR1 (ERa), FAS (TNFRSF6), GAA, HTT, IFNG, IGF1, INS, IRGM, MAP1LC3A, MAPK8 (JNK1), NFKB1, PIK3C3 (VPS34), RPS6KB1, SNCA, SQSTM1, TNF, TP53, ULK1
Necrosis	ATP6V1G2, BMF, C1orf159, CCDC103, COMMD4, CYLD, DEFB1, DENND4A, DPYSL4, EIF5B, FOXI1, GALNT5, GRB2, HSPBAP1, JPH3, KCNIP1, MAG, OR10J3, PARP1 (ADPRT1), PARP2, PVR, RAB25, S100A7A, SPATA2, SYCP2, TMEM57, TNFRSF1A, TXNL4B.

Source: Qiagen, Frederick, MD, EUA.

Table S2. shRNA sequences used for CHOP knockdown in K562 cells.

**Lentiviral vector**	**Sequence**
TRCN0000007263	CCGGGCCAATGATGTGACCCTCAATCTCGAGATTGAGGGTCACATCATTGGCTTTTT
TRCN0000364328	CCGGCTGCACCAAGCATGAACAATTCTCGAGAATTGTTCATGCTTGGTGCAGTTTTTG
TRCN0000364393	CCGGTGAACGGCTCAAGCAGGAAATCTCGAGATTTCCTGCTTGAGCCGTTCATTTTTG
